# Re: Implication of low‐dose sacubitril/valsartan

**DOI:** 10.1002/clc.23560

**Published:** 2021-02-11

**Authors:** Amitabh C. Pandey, James Thomas Heywood

**Affiliations:** ^1^ Division of Cardiology Scripps Prebys Cardiovascular Institute, Scripps Clinic La Jolla California USA; ^2^ Scripps Research Translational Institute, The Scripps Research Institute La Jolla California USA

1

To the Editor

We thank Imamura et al. for their interest in our recent study and appreciate the opportunity to address their comments.^1^ Dr. Imamura suggested baseline differences in three groups in our study and should be corrected.^2^ While we agree that there are small differences in the groups, these serve as a reminder of the real‐world differences in the cohort of patients labeled at having heart failure with reduced ejection fraction (HFrEF). Because of the limited number of patients in our trial, adjusting for differences would not make any finding statistically significant. A much larger trial would be necessary to accomplish this. Our study serves more as a pilot/proof of concept rather than an advanced‐stage clinical trial. The results of our study show that, despite the lower blood pressure of some of the cohorts, these cohorts are able to be started on angiotensin‐neprilysin receptor antagonist (ARNI) therapy and are even titrated up to higher doses in some cases. In addition, Dr. Imamura suggests that the use of a control group would have further aided our study. However, a true control group—lacking any guideline‐based medical therapy—would have given rise to an ethical dilemma given the efficacy of Guide line directed medical therapy (GMT). Furthermore, Prospective Comparison of ARNI with ACEI to Determine Impact on Global Mortality and Morbidity in Heart Failure (PARDIGM‐HF) already has shown the benefits of ARNI compared to monotherapy with angiotensin‐converting enzyme inhibitors or angiotensin receptor blockers.^3^ Finally, even Comparison of Sacubitril‐Valsar‐tan versus Enalapril on Effect on NT‐proBNP in Patients Stabilized from an Acute Heart Failure Episode (PIONEER) showed significant reduction in NT‐proBNP levels in hospitalized patients, making a comparison group without ARNI therapy lack significant additional value.^4^ In addition, all patients had a baseline NT‐proBNP level, which was compared to the final level. The baseline level was in the absence of ARNI therapy, on maximally tolerated guideline‐based therapy, and utilization of ARNI therapy—at the dose response our study showed—resulted in a significant decrease in NT‐proBNP levels.

While ARNI therapy even at lower doses does appear to be promising in allowing more patients to tolerate therapy and has a correlation with benefits, including lower NT‐proBNP levels, reduction in diuretic dosing, and stable renal function, we do agree that future prospective trials will be able to provide more insight into the benefits and mechanisms of ARNI therapy for HFrEF patients.

## DISCLOSURE OF INTERESTS

Amitabh C. Pandey is funded by NIH NCATS CTSA Award to Scripps Research Translational Institute. James Thomas Heywood serves as a speaker and consultant of Novartis.
